# Decompressive craniectomy for herpes simplex encephalitis complicated by frank intracerebral hemorrhage: a case report and review of the literature

**DOI:** 10.1186/s12883-018-1181-6

**Published:** 2018-10-23

**Authors:** Yoon Hwan Byun, Eun Jin Ha, Sang-Bae Ko, Kyung Hyun Kim

**Affiliations:** 1 Department of Neurosurgery, Seoul National University Hospital, 101 Daehak-ro, Jongno-gu, Seoul, 03080 Republic of Korea; 2 Department of Neurology, Seoul National University Hospital, 101 Daehak-ro, Jongno-gu, Seoul, 03080 Republic of Korea

**Keywords:** Herpes simplex encephalitis, Intracerebral hemorrhage, Surgical decompression

## Abstract

**Background:**

Herpes simplex encephalitis is the most common type of sporadic encephalitis worldwide. Frank intracerebral hemorrhage complicating the disease course in herpes simplex encephalitis patients is rare, especially cases where surgical decompression is necessary. Here, we report a previously healthy female with herpes simplex encephalitis who underwent surgical decompression due to temporal lobe hemorrhage.

**Case presentation:**

A previously healthy 34-year-old Korean female presented with fever, myalgia and severe headache. Brain MRI showed a high T2 signal intensity change and diffuse swelling of the right temporal lobe. Polymerase chain reaction testing of the cerebrospinal fluid confirmed the presence of herpes simplex virus 1. The patient was admitted for close observation and intravenous acyclovir. On hospital day 3, she had a sudden onset of vomiting and severe headache. Brain CT showed frank temporal lobe hemorrhage. Despite aggressive medical treatment, she became increasingly drowsy. Ultimately, she underwent emergency right decompressive craniectomy, expansile duraplasty and intracranial pressure monitor insertion. The patient recovered fully without any neurological deficits or neuropsychological problems. She was discharged after completion of 2 weeks of acyclovir and returned 2 months later for cranioplasty.

**Conclusions:**

Patients with severe herpes simplex encephalitis complicated by intracerebral hemorrhage or malignant cerebral edema should undergo aggressive medical treatment. Surgical decompression should also be actively considered in these severe cases to prevent further neurological deterioration.

## Background

Herpes simplex encephalitis (HSE) is a severe viral infection of the human central nervous system caused by either herpes simplex virus (HSV) 1 or 2. This infection is known to be the most frequent form of sporadic encephalitis worldwide, accounting for 10–20% of all cases [1]. Patients with HSE frequently present with fever, headache, seizure or focal neurological deficits [2]. Encephalitis characterized by cerebral edema and hemorrhagic necrosis typically involves the temporal lobe, which is best visualized by magnetic resonance imaging (MRI) of the brain [3, 4]. The diagnosis of HSE is confirmed by positive polymerase chain reaction (PCR) results for HSV DNA in the cerebrospinal fluid (CSF) [5]. The fatality rate of HSE has been reduced to 5–15% from 70% with the development of viral therapy [6]. However, even after initiation of acyclovir treatment, cerebral edema may progress and cause mass effects, increased intracranial pressure (ICP) and uncal herniation [7]. Frank intracerebral hematoma formation complicating the disease course of HSE is reported to be rare. We report a case of delayed temporal lobe hemorrhage in an immunocompetent patient with HSE who ultimately underwent surgical decompression.

## Case presentation

**Fig. 1:**
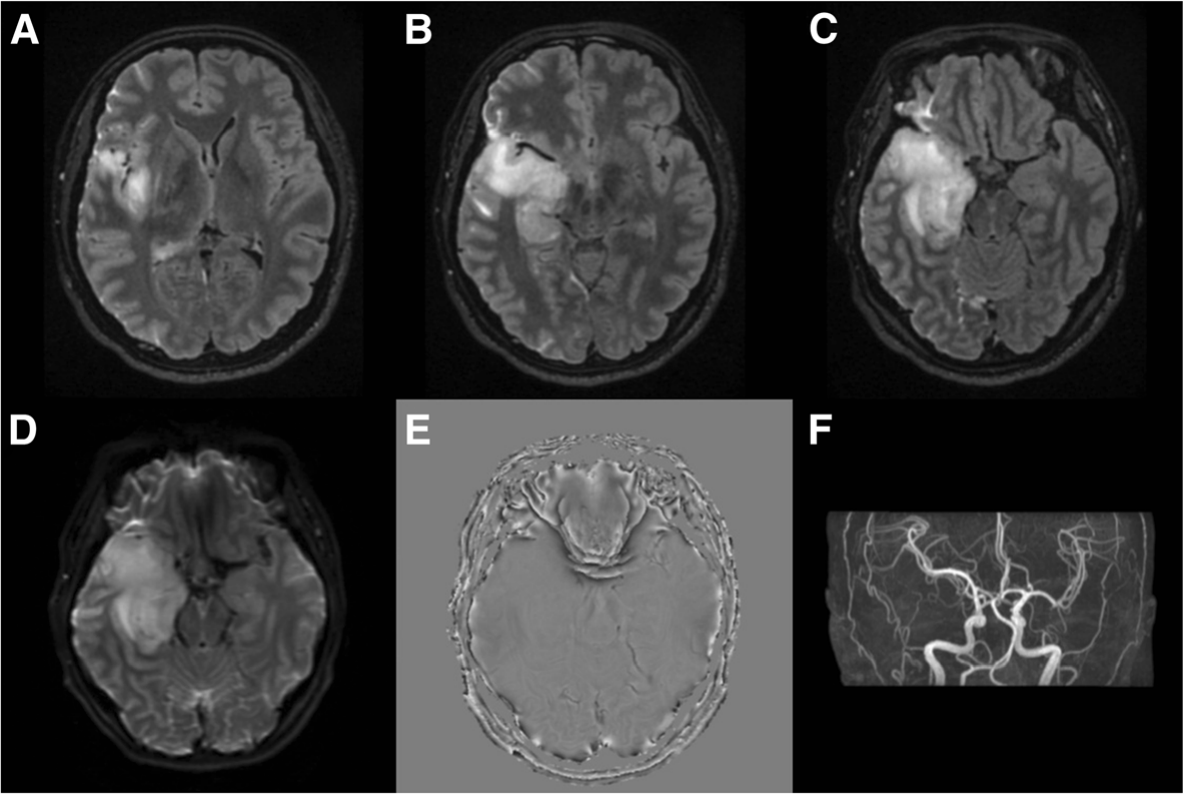
Initial brain MRI (2017.12.15). T2 weighted image shows diffuse swelling and high signal intensity changes of the right (**a**) insula (**b**) hippocampus (**c**) temporal lobe. **d** Diffusion weighted image (DWI) shows diffusion restriction of the right temporal lobe lesion (**e**) Susceptibility weighted angiography (SWAN) shows no evidence of hemorrhage (**f**) Brain MR Angiography shows no steno-occlusive lesion or aneurysm in intracranial vessels

A previously healthy 34-year-old Korean female was admitted to a regional hospital for fever, myalgia and severe headache that had started 2 weeks ago. Brain MRI showed a high T2 signal intensity change and diffuse swelling of the right temporal lobe, insula and hippocampus (Fig. 1). Lumbar puncture showed lympho-dominant pleocytosis. The patient was started on intravenous (iv) dexamethasone and acyclovir under a high suspicion of viral encephalitis.

**Fig. 2:**
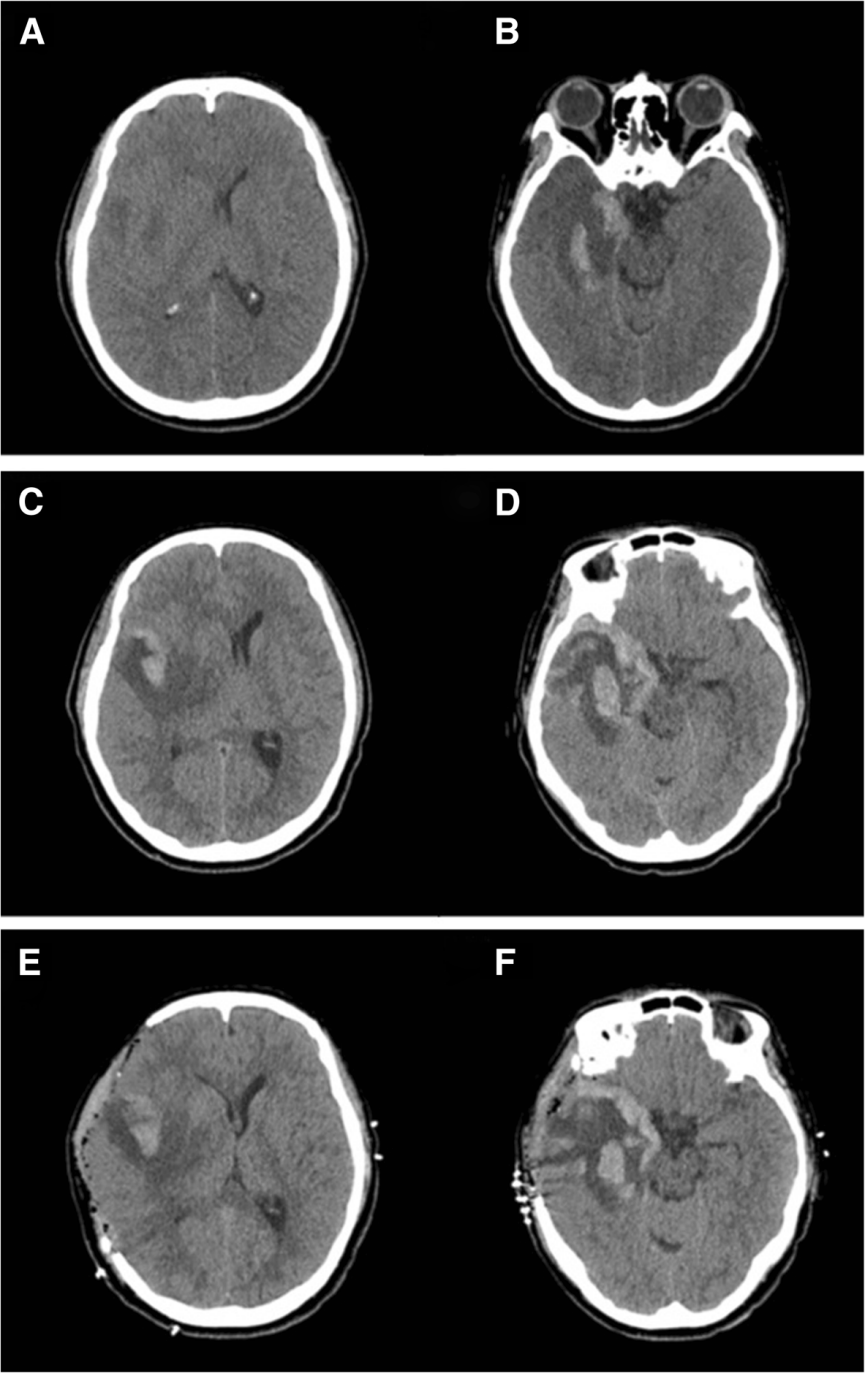
Serial follow up noncontrast brain CT. **a**, **b**: Brain CT taken on the day of admission to our institution (2017.12.19) shows a hemorrhagic transformation of the right temporal lobe lesion. **c**, **d**: Brain CT taken on day 3 of hospitalization (2017.12.22) shows an increased amount of temporal lobe hemorrhage and a leftward shift in the midline. **e**, **f**: Postoperative brain CT (2017.12.23) shows an improvement in midline shift with no significant increase of temporal lobe hemorrhage

She was transferred to our institution 4 days later due to persistent headache despite treatment. A noncontrast computed tomography (CT) brain scan taken at our institution showed a hemorrhagic transformation of the right temporal lobe, which was not observed on the initial MRI (Fig. 2a, b). Follow-up lumbar puncture showed 510 white cells per mm^3^ (82% lymphocytes), 144 mg/dL protein and 61 mg/dL glucose. CSF culture studies were negative for bacteria, fungi and tuberculosis. PCR of the CSF confirmed the presence of HSV1. The patient was free of neurological symptoms, with a Glasgow Coma Scale of E4M6V5, and was admitted for close observation and continuation of iv acyclovir. Corticosteroid treatment was discontinued upon her admission. On day 3 of hospitalization, the patient presented with a sudden onset of vomiting and severe headache. Brain CT showed an increased amount of temporal lobe hemorrhage and a leftward shift in the midline (Fig. 2c, d). Mannitol was administered but did not seem to have a significant effect. The patient became increasingly drowsy, and her right pupil became dilated. She underwent emergency right decompressive craniectomy, expansile duraplasty and ICP monitor insertion. Postoperative brain CT showed alleviation of midline shifting (Fig. 2d, e). The patient recovered fully 5 days after the surgery. Apart from mild intermittent headache and dizziness, she did not show any other significant clinical symptoms, including neuropsychological problems. There were no significant neurologic deficits upon neurological examinations performed by the attending neurosurgeon and neurologist. The patient was discharged after completion of 2 weeks of acyclovir and returned 2 months later for cranioplasty. She was followed up 3 more times after cranioplasty. She was stable, without any neuropsychological problems or neurologic deficits, and was able to successfully return to work as a public official.

## Discussion and conclusions

Petechial cortical hemorrhages in HSE are common. However, frank hematoma formation in HSE is rare, especially in cases requiring surgical decompression. To the best of our knowledge, only 8 similar adult cases [8–14] have been reported in the English literature; these cases are summarized in Table 1. Acyclovir was started promptly upon admission in all cases, and the mean interval from admission to hematoma formation was 8 days. The exact mechanism of hemorrhage has not been found, but a few hypotheses have been proposed. These hypotheses include the rupture of small vessels due to either vasculitis or transient hypertension caused by increased ICP [15].

**Table 1:** Patients who underwent surgical decompression for HSE complicated by intracerebral hemorrhage

Reference	Age/Sex	Initial clinical presentation	Admission to hemorrhage lapse (days)	New symptoms associated with hemorrhage	Location	Treatment				Outcome	Other medical condition
						Medical			Surgical		
						Acyclovir	Mannitol	Steroid			
Rodriguez-Sainz et al. [8]	45/F	fever, headache, incoherent speech	9	lt third nerve palsy, rt. hemiparesis, severe aphasia	lt temporal	ACV 10 mg/kg 8 h x 21d	o	x	DC + HE	rt hemiparesis, motor aphasia	hepatitis C
Yan [9]	37/M	fever, headache, bizarre behavior, hallucination, memory problem	8	altered mental status, anisocoric pupil	lt temporal	ACV 10 mg/kg/8 h x 18d	x	x	TL	full recovery	
	48/F	headache, altered mental status, hemianopsia	10	rt hemiparesis, anisocoric pupil	lt temporal	ACV 10 mg/kg/8 h x 12d	x	x	DC + TL	mild stuttering	
Counsell et al. [10]	32/M	fever, headache, seizure	10	altered mental status, partial rt. third nerve palsy	rt fronto-temporal	ACV 10 mg/kg/d x 3d +10d (post-operative)	x	o	TL	full recovery	
Jabbour et al. [11]	27/M	fever, headache, seizure	9	altered mental status, partial rt. third nerve palsy	rt temporal	ACV 10 mg/kg/12 h (duration not specified)	x	x	HE	full recovery	iv heparinization for pulmonary embolism
Li et al. [12]	56/M	seizure, impairment in memory	6	altered mental status	lt temporal	ACV x 28d	x	x	DC + HE	impairment in word finding, memory impairment	HIV (+)
Lo et al. [13]	46/M	fever, headache, vomiting, confusion	6	rt hemiparesis, anisocoric pupil	lt temporal	ACV 10hg/kg/d x 36d	x	o	TL + HE + delayed DC	full recovery	
Mueller et al. [14]	40/F	fever, headache, nausea, photophobia	7	altered mental status, lt hemiparesis	rt temporal	ACV x 21d	x	x	DC + TL	not specified	
Current case	34/F	fever, headache, myalgia	7	altered mental status, anisocoric pupil	rt temporal	ACV x 14d	o	o	DC	full recovery	

*ACV* acyclovir, *Lt* Left, *Rt* Right, *DC* decompressive craniectomy, *HE* hematoma evacuation, *TL* temporal lobectomy, *HIV* human immunodeficiency virus

In HSE patients, administration of acyclovir should be initiated immediately to prevent further viral replication in the brain [16]. Acyclovir significantly improves the outcomes achieved by HSE patients, and late initiation of viral treatment can lead to significant morbidity and mortality. The Infectious Disease Society of America (IDSA) recommends 2–3 weeks of intravenous acyclovir at 10 mg/kg/8 h depending on the clinical course [17]. Some HSE patients present with atypical symptoms and/or normal initial CSF analysis, which may hinder physicians from reaching the correct diagnosis. Repeated clinical and laboratory tests are recommended when encephalitic syndromes are suspected, and acyclovir should be considered in these patients even before proven diagnosis [18].

Any worsening of initial symptoms or manifestation of new neurologic deficits despite adequate viral treatment, especially during the second week of admission, warrants the need for neuroimaging study [8]. Aggravation of cerebral edema and formation of new intracerebral hemorrhage should be ruled out. In addition, acyclovir resistance and acyclovir side effects should be considered in the differential diagnosis.

Medical treatment to control increased ICP due to cerebral edema or hematoma includes hyperventilation, corticosteroids and hyperosmolar therapy. The use of corticosteroids in HSE is controversial due to its potential to increase viral replication, resulting in further cell damage [19]. However, previously reported studies generally support the use of corticosteroids in severe cases of HSE after the initiation of antiviral therapy [20]. Corticosteroids may benefit these patients by inhibiting the inflammatory cascades that lead to vasogenic edema in the latter part of the disease course. There is no concrete evidence regarding the use of mannitol or hypertonic saline in HSE, and the use of hyperosmolar therapy to reduce ICP in these situations is extrapolated from traumatic brain injury guidelines [21]. However, we postulate that hyperosmolar therapy may help alleviate cytotoxic edema caused by viral replication early in the course of HSE.

There is a lack of literature concerning the utilization of surgical decompression, such as decompressive craniectomy or temporal lobectomy, in patients with severe HSE. Nonetheless, presently available data generally support the use of surgical decompression in viral encephalitis with severe life-threatening neurological symptoms [22]. Our patient underwent surgical decompression due to neurological deterioration despite aggressive medical treatment and was able to recover fully without neurological deficits. We support the use of surgical decompression in severe HSE cases complicated by malignant cerebral edema or intracerebral hemorrhage.

In conclusion, frank intracerebral hemorrhage in HSE is a rare complication. This occurrence can cause neurological deterioration due to a mass effect or increased ICP. Neuroimaging studies should be conducted in HSE patients who show unchanged or worsened symptoms even after the initiation of acyclovir. Patients with severe HSE complicated by intracerebral hemorrhage or malignant cerebral edema should undergo aggressive medical treatment. Surgical decompression should also be actively considered in such severe cases to prevent further neurological deterioration.

## Abbreviations

CSF: Cerebrospinal fluid
CT: Computed tomography
HSE: Herpes simplex encephalitis
HSV: Herpes simplex virus
ICP: Intracranial pressure
MRI: Magnetic resonance imaging
PCR: Polymerase chain reaction
